# PGP-Bacterium *Pseudomonas protegens* Improves Bread Wheat Growth and Mitigates Herbicide and Drought Stress

**DOI:** 10.3390/plants11233289

**Published:** 2022-11-29

**Authors:** Margarita Bakaeva, Sergey Chetverikov, Maksim Timergalin, Arina Feoktistova, Timur Rameev, Dar’ya Chetverikova, Aliya Kenjieva, Sergey Starikov, Danil Sharipov, Gaisar Hkudaygulov

**Affiliations:** Ufa Institute of Biology of Ufa Federal Research Centre of Russian Academy of Sciences, 450054 Ufa, Russia

**Keywords:** wheat, drought, herbicide, plant growth promoting bacteria, phytohormone, yield

## Abstract

The reaction of plants to simultaneous stress action and treatment with biological stimulants still remains poorly studied. Laboratory and field experiments have been conducted to study the growth and yield of bread wheat (*Triticum aestivum* L.) of the variety Ekada 113; stress markers and quantitative ratios of phytohormones in plants under insufficient soil moisture; the effects of spraying with herbicide containing 2,4-D and dicamba and growth-stimulating bacterium *Pseudomonas protegens* DA1.2; and combinations of these factors. Under water shortage conditions, spraying plants with Chistalan reduced their growth compared to non-sprayed plants, which was associated with inhibition of root growth and a decrease in the content of endogenous auxins in the plants. Under conditions of combined stress, the treatment of plants with the strain *P. protegens* DA1.2 increased the IAA/ABA ratio and prevented inhibition of root growth by auxin-like herbicide, ensuring water absorption by the roots as well as increased transpiration. As a result, the content of malondialdehyde oxidative stress marker was reduced. Bacterization improved the water balance of wheat plants under arid field conditions. The addition of bacterium *P. protegens* DA1.2 to the herbicide Chistalan increased relative water content in wheat leaves by 11% compared to plants treated with herbicide alone. Application of the bacterial strain *P. protegens* DA1.2 increased the amount of harvested grain from 2.0–2.2 t/ha to 3.2–3.6 t/ha. Thus, auxin-like herbicide Chistalan and auxin-producing bacterium *P. protegens* DA1.2 may affect the balance of phytohormones in different ways. This could be the potential reason for the improvement in wheat plants’ growth during dry periods when the bacterium *P. protegens* DA1.2 is included in mixtures for weed control.

## 1. Introduction

In the field, agricultural crops are exposed to various negative factors. Drought is one of the main reasons for the decrease in yields and quality of agricultural products, especially in regions with unstable rainfall. Herbicides are actively used to control weeds in crops of cultivated cereals. Death of most plant tissues treated with 2,4-D and other similar herbicides is caused by oxidative stress induced by high ROS production. Christoffoleti et al. [1] summarizes the results of several studies in the review and describes this process. Synthetic auxin first acts on plasma membrane-activating ABP1, altering the cytoskeleton and, consequently, reducing the peroxisomes’ antioxidative defense. Next, the second process acts by the TIR/AFB ubiquitination route, in which ABA and ethylene biosynthesis genes are activated, leading to ROS overproduction. *Triticum aestivum* is the non-target species for these herbicides. However, their accumulation in the soil can also lead to stress in wheat plants [2,3]. Kumar and Singh [4] made an attempt to review the harmful effects of 2,4-D on wheat plants. They give examples of various manifestations of the toxic effect of 2,4-D, and express the opinion that these manifestations depend on wheat genotypes. Stressors often act on plants simultaneously. For example, herbicidal weeding may be required during dry field seasons. It has been shown that the reaction of plants to combined abiotic stresses cannot be deduced from the study of plants which were subjected to each stress separately [5]. Unique features have been discovered when studying various plant processes and systems: genes and enzymes [6], signaling molecules [7], photosynthesis [8], and oxidative stress [9]. Benevenuto et al. [10] found that drought and the herbicide glyphosate enhance each other’s influence on the metabolic profile of genetically modified soybeans. The herbicide Serrate^®^ (Syngenta, Basel, Switzerland) has the active ingredients pyroxsulam (7.5%) and clodinafop (20%), and provoked an additional load of biochemical components associated with oxidative stress under drought conditions [3]. Together, herbicide and drought treatments increased oxidative stress markers, activated enzymatic and non-enzymatic antioxidant defense, [3] and inhibited photosynthetic performance [11].

Microorganisms have significant potential to obtain effective and environmentally friendly plant growth regulators and antidotes that mitigate herbicidal stress on their basis. The potential advantage of bacteria is their versatility. They are simultaneously capable of stimulating growth; suppressing phytopathogens; accelerating decomposition of pesticides in the soil; and penetrating to the rhizosphere biome, which prolongs their effect [12,13,14]. How bacteria help plants during drought is fairly well-understood and has been well-summarized. Conventionally, two types of positive effects of plant growth-promoting (PGP) bacteria can be distinguished: a direct effect on the mechanisms of adaptation of plants to drought, and indirect influence through the creation of favorable conditions for growth. Bacteria can regulate the adaptive reactions of plants through the production of phytohormones, osmolytes, antioxidants, volatile compounds, exopolysaccharides, and 1-aminocyclopropane-1-carboxylate deaminase [15,16]. These metabolites contribute to the absorption and retention of water by plant tissues, counteract oxidative stress, and limit production of stress hormones. Bacteria create favorable conditions for plant growth by suppressing phytopathogens and converting nitrogen, phosphorus, and trace elements into a form available to plants.

The use of PGP bacteria to reduce herbicidal stress has been studied relatively recently, and has been reviewed in a small number of publications [17,18,19,20]. In the experiments described therein, an improvement in the growth of agricultural plants and mitigation of herbicidal stress after spraying with bacterial solution were observed. Works explaining the mechanisms of herbicide stress mitigation are also few, and those few are focused primarily on microbial endophytes [21,22]. They suggest that endophytes can directly contribute to herbicide detoxification through their ability to metabolize xenobiotics. In addition, they are exploring the paradigm that microbes can ‘prime’ resistance mechanisms in plants in such a way that they enhance herbicide tolerance by inducing the host stress responses, in order to withstand the downstream herbicide-induced toxicity. We found no studies examining the effect of PGP bacteria on hormone levels in cereals when they were treated with herbicides in combination with drought. It would be especially interesting to investigate this effect in relation to herbicides with a phytohormonal action (2,4-D, dicamba).

In this work, we studied the effects of spraying wheat plants (*Triticum aestivum* L.) with the growth-stimulating bacterium *P. protegens* DA1.2. This was performed under conditions of combined stress of moisture deficiency as well as the presence of a herbicide Chistalan based on synthetic auxins. The purpose of the work was to obtain new data on growth parameters, the balance of phytohormones, and the yield of bread wheat variety Ekada 113 under a deficit of soil moisture after treatment with the auxin-like herbicide Chistalan and the plant growth-promoting bacterial strain *P. protegens* DA1.2.

## 2. Results

### 2.1. Plant Weight

**Figure 1:**
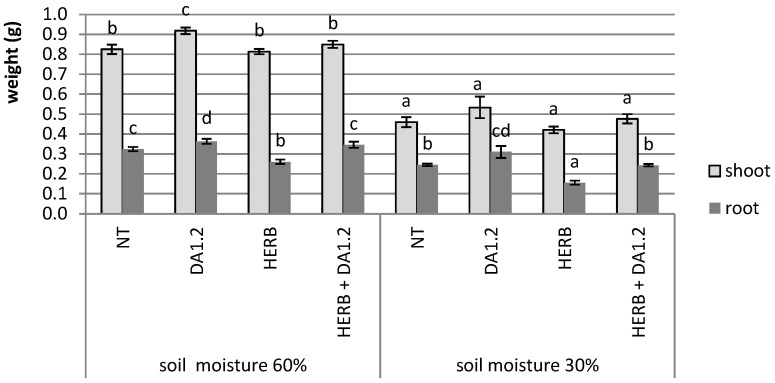
The weight of wheat plant on the 14th day after treatment. NT—no treatment was performed; DA1.2—bacterial treatment with *Pseudomonas protegens* DA1.2; HERB—herbicide. Statistically different means are indicated by different letters (*p* ≤ 0.05, *n* = 50).

The results of weighing the roots and shoots of wheat which had grown under different model conditions are shown in Figure 1. Some trends can be distinguished. A strong trend was a 40–55% decrease in the wet weight of shoots under drought stress. A less clear trend was a 23% decrease in the weight of wheat roots after herbicide Chistalan spraying at normal moisture, and a 36% decrease at 30% soil moisture. Mixing an auxin-like herbicide with a bacterial culture, on the contrary, activated the growth of wheat roots compared to plants untreated with bacteria of the strain *P. protegens* DA1.2. Coordinated stimulation of root and shoot growth by microorganisms was observed only under favorable soil moisture, without application of herbicide.

### 2.2. Characteristics of Water Relations

**Figure 2:**
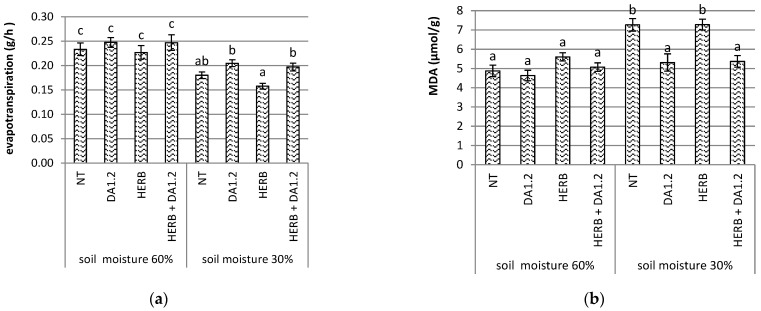
Indicators of wheat plants in pots on the 7th day after treatment: (**a**) evapotranspiration per one plant, *n* = 25; (**b**) the amount of malondialdehyde in leaves, *n* = 9. NT—no treatment was performed; DA1.2—bacterial treatment with *Pseudomonas protegens* DA1.2; HERB—herbicide treatment. Statistically different means are indicated by different letters (*p* ≤ 0.05).

Our calculations of the amount of water evaporated from each container with plants showed that when wheat plants were provided with sufficient water, neither spraying with herbicide Chistalan nor treatment with bacterium *Pseudomonas protegens* DA1.2 significantly affected this indicator (Figure 2). However, at 30% soil moisture, in containers with herbicide, there was a tendency of a decrease in evapotranspiration. When bacterial culture was added to the Chistalan, on the contrary, there was a tendency of increased evapotranspiration. The difference between the evapotranspiration of pots sprayed with herbicide and a mixture of herbicide with bacterium *P. protegens* DA1.2 reached 25%, and was shown to be reliable.

### 2.3. Determination of Lipid Peroxidation

Malondialdehyde (MDA) is a product of lipid peroxidation. Its concentration in cells increases in the case of accumulation of active oxygen, and this indicator is used as a marker of oxidative stress in the study of many species of living organisms. In wheat plants grown in pots, the amount of MDA in the leaves increased with a shortage of soil water, as well as with a combination of artificial drought with herbicide Chistalan spraying (Figure 2). The addition of bacterial cultures to these two factors led to a decrease in the amount of MDA in the leaves, so that it did not differ significantly from the control in the absence of drought or any treatments.

### 2.4. Phytohormones

**Figure 3:**
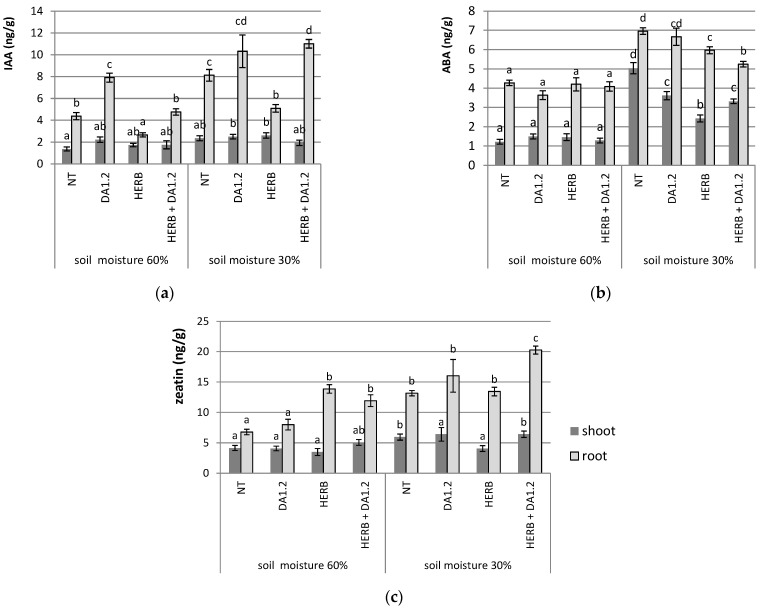
The amount of phytohormones in wheat leaves and roots: (**a**) indolylacetic acid; (**b**) abscisic acid; (**c**) zeatin. NT—no treatment was performed; DA1.2—bacterial treatment with *Pseudomonas protegens* DA1.2; HERB—herbicide treatment. Statistically different means are indicated by different letters (*p* ≤ 0.05, *n* = 6).

Concentration of the phytohormone indolylacetic acid (IAA) in wheat shoots fluctuated slightly in different versions of the experiment (Figure 3). A significant increase in its concentration in the leaves was shown only for plants sprayed with Chistalan which experienced a lack of soil moisture. In plant roots, dry conditions caused the accumulation of IAA in all cases except for individual herbicide treatment. The amount of phytohormone increased by 90–150%.

With sufficient soil moisture, the use of herbicide contributed to a 1.6-fold decrease in the amount of IAA in wheat roots. The concentration of IAA in the roots of plants positively and most clearly changed in response to the presence of microorganisms. In our experiment, the maximum accumulation of IAA in the roots was recorded precisely after the addition of bacterium *Pseudomonas protegens* DA1.2 under arid conditions.

The abscisic acid (ABA) content changed primarily in response to the shortage of water for wheat plants. In arid conditions, it increased both in the roots and in the leaves compared to the control. The introduction of both microorganisms and the herbicide Chistalan rather counteracted the synthesis of ABA by plants under drought conditions. Changes in the amount of zeatin were observed mainly in the roots, increasing by 80–100% under the conditions of poor watering of the wheat and plant treatment with herbicide. However, the growth of this indicator was most noticeable when three factors (drought, herbicide, and bacterium) were acting simultaneously.

### 2.5. The Effect of Treatments on Phytohormones and Yield of Bread Wheat in the Field

Since the bacterium *P. protegens* DA1.2 is resistant to Chistalan and also metabolizes it, the microbiological sowing of the soil on a selective mineral medium, containing the herbicide Chistalan as the sole source of carbon and energy, was applied. No isolates were obtained from soil samples without *P. protegens* DA1.2 treatment using this nutrient medium. The sensitivity of the method was 10^2^ CFU/g, and the number of CFUs that metabolize Chistalan was below this value. The number of CFUs isolated from soils treated with only the bacterium *P. protegens* DA1.2 and its combination with Chistalan were (1.3 ± 0.2) × 10^3^ CFU g^−1^ and (5.5 ± 0.4) × 10^3^ CFU g^−1^. The morphology of colonies and cells did not differ from those of bacterium *P. protegens* DA1.2.

Thus, the data obtained in the laboratory indicated that growth-stimulating culture could contribute to the normalization of water uptake by plants under arid conditions. To test this thesis in the field, during the dry periods of 2020 and 2021, the relative water content (RWC) was measured in wheat leaves 10 days after their treatment with auxin herbicide or a mixture of herbicide with bacteria of strain *P. protegens* DA1.2. After the application of Chistalan, RWC was 19% lower than in the untreated plots (Table 1). RWC was the same in the plots treated with the mixture of bacterium *P. protegens* DA1.2 and Chistalan and in the untreated plots. 

The use of the two-component herbicide Chistalan contributed to a decrease in the amount of ABA in shoots and IAA in roots. Wheat plants responded to spraying with the bacterium *Pseudomonas protegens* DA1.2 by increasing the amount of IAA in the roots. 

The number of weeds on the plots with different treatments was as follows (plants m^−2^): without treatment—37 ± 3; only bacterial culture—33 ± 4; only herbicide—1.5 ± 0.3; herbicide and bacterial culture—1.8 ± 0.4. Weeds belonged to the species *Cirsium arvense* L. and *Convolvulus arvensis* L.

**Figure 4:**
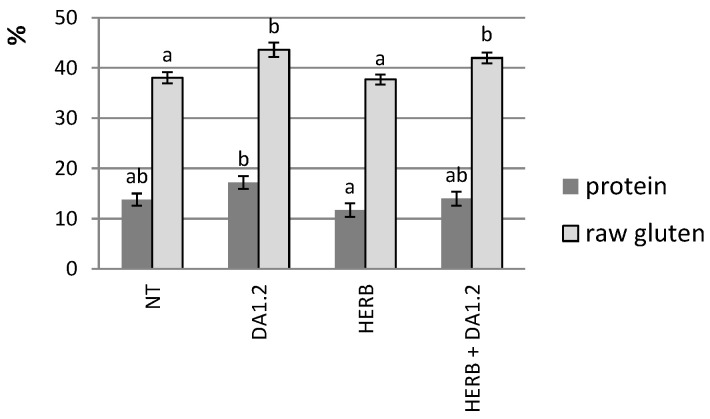
The percentage of protein (*n* = 3) and raw gluten (*n* = 6) in the average sample of grain harvested in 2020–2021. NT—no treatment was performed; DA1.2—treatment with *Pseudomonas protegens* DA1.2; HERB—herbicide treatment. Statistically different means are indicated by different letters (*p* ≤ 0.05).

At the end of the agricultural season, the inclusion of the bacterium *P. protegens* DA1.2 in agricultural technology contributed to an increase in the yield, which reached 1520 kg/ha, compared to spraying with herbicide only. The amount of straw was also increased. Among wheat yield indicators, the weight of 1000 grains did not depend on the type of treatment, while the number of productive stems responded markedly to the use of both the bacterial strain *P. protegens* DA1.2 and the herbicide Chistalan. The number of productive stems increased by 32% after treatment with bacterium *P. protegens* DA1.2, and by 50% after mixed treatment. Spraying with the herbicide Chistalan had a positive effect on the spike in the number of grains. Obviously, the better provision of plants with water during the dry period, along with other beneficial properties of bacterium *P. protegens* DA1.2, led to an increase in the size of wheat plants and its yield. Evaluation of the accumulation of protein components in the grain gave mixed results (Figure 4). There was no strong difference between the variants with and without bacterial treatment in the amount of protein calculated from total nitrogen, while different types of treatment affected the amount of raw gluten. The trend of positive influence of the bacterium *P. protegens* DA1.2 on this indicator was clearly noticeable.

## 3. Discussion

Drought adversely affects grain crops. Plants implement a complex set of adaptations in order to survive a drought. They involve the inhibition of shoot growth, a decrease in the area of leaves, an increase in the ratio of roots and shoots, the stomatal closure, the accumulation of osmotically active substances, and much more [23,24]. These adaptive responses reduce the evaporating surface of the leaves and contribute to drought survival. Some of the morphological changes that we observed in wheat plants treated with the auxin-like herbicide Chistalan contradicted this typical strategy. The inhibition of root growth is not consistent with the normal adaptation of plants to arid conditions, since it reduces the availability of soil water for plants. The trends of decreasing transpiration and relative water content in the leaves of herbicide-sprayed plants can be explained by the lower mass of their root system.

The data found in the literature regarding the effect of auxin-like herbicides on the morphology of non-target plants are quite contradictory. There are works that agree with the results we have obtained about the negative effect of the herbicide 2,4-D on root growth [25,26]. In the article in [27], on the contrary, the use of 2,4-D attenuated salinity-induced toxicity in rice cultivars. The observed differences may be related to the characteristics of the plant varieties used and the dose of herbicide that was applied to the plants. It is well known that many herbicides are toxic to plants in high doses, and at lower doses, they act as growth regulators [28]. The uptake of 2,4-D by the plant may be reduced under certain soil conditions, for example, with salinity or severe drought. When wheat plants were additionally treated with bacterium *P. protegens* DA1.2, the herbicide’s inhibitory effect on root growth was not detected. Apparently, the activation of growth of the root system increased its absorption capacity. In accordance with this, the water deficit in the leaves decreased, and transpiration became more active.

When attempting to explain the observed effects, the hypothesis of a change in the hormonal system of plants was logical, since the herbicide was an artificial phytohormone and the bacteria were capable of synthesizing plant growth regulators. The balance of phytohormones plays an important role in the plant’s protective mechanisms during drought. The well-studied reaction caused by a deficit in soil moisture is the activation of ABA synthesis [29]. ABA controls a number of reactions (stomatal closure, synthesis of osmoprotectors, and antioxidant systems) that help plants to survive under water shortage. On the other hand, the closure of stomata caused by ABA triggers a number of negative consequences: a decrease in the efficiency of photosynthesis, and oxidative and thermal stresses. Under moisture deficits, the amount of ABA in the leaves decreased by 34% and 52% after bacterial introduction and spraying with the herbicide Chistalan, respectively. This was intended to ameliorate the conditions for photosynthesis and growth. In fact, growth was observed only in pots treated with bacterium *P. protegens* DA1.2. Apparently, in this case, the growth potential was reinforced by the influx of water and nutrients from the roots. The increase in the ratio of the mass of the root system to the mass of shoots also has an adaptive character, and is obviously associated with the accumulation of a root growth activator, IAA. In this aspect, the reaction of wheat plants observed in our experiment was typical. As a synthetic auxin, 2,4-D interacts with phytohormones and can disturb their balance. In our experiment, the most pronounced reaction of the hormonal system of plants to treatment with the herbicide Chistalan was a decrease in the endogenous IAA in the roots. This may be due to the fact that an excess of auxins can inhibit the synthesis and transport of endogenous auxin, and can promote its oxidation [30]. The tendency of reduced endogenous auxin and decreased root mass in herbicide-treated plants was similar with both sufficient and deficient hydration. A poorly developed root system can cause insufficient water uptake by the plant. This can be a critical factor for its normal growth under drought conditions.

The effect of *Pseudomonas protegens* DA1.2 on the plants’ morphology consisted of an increase in root mass, mainly due to adventitious and lateral root growth. The obtained data on phytohormones allow us to explain the observed effect. In the presence of bacteria, the amount of IAA in the roots increased, which was especially noticeable under low soil moisture. The capacity to stimulate the formation of additional roots is a well-known effect of this hormone. Apparently, the synthesis of IAA by bacteria contributed to the accumulation of this hormone in the roots of wheat. The close relationship between the ability of rhizosphere bacteria to produce IAA and its contents in plant tissues has previously been reported by other authors [31,32].

The ratio of auxins to cytokinins is an important factor in cell division and differentiation of plant tissues. The crosstalk between these hormones largely determines the development and architecture of the roots [33,34]. The shift of this ratio towards cytokinins contributes to the inhibition of root system growth [35]. Thus, the increase in the amount of cytokinins observed in the herbicide-treated variants of the experiment, although the amount of IAA was reduced, may be one of the reasons for the low root mass. In some tissues, synthetic exogenous auxin 2,4-D is also able to activate zeatin biosynthesis [36]. In contrast to the use of the herbicide Chistalan after bacterial treatment, IAA and cytokinins accumulated in the roots in parallel. Thus, the ratio of IAA/cytokinins became closer to what was observed in plants untreated with Chistalan under drought conditions. The de-stabilizing effect of the herbicide on this ratio was significantly mitigated.

Despite numerous evidence confirming the positive effect of PGP bacteria on plant growth under controlled conditions, their use in the open field is not always effective [37,38]. The displacement of introduced bacteria from the soil microbiome by native microorganisms and changeable weather lead to the effects detected in laboratory conditions not being recorded in the field. Therefore, field experiments are very desirable in the studies involving PGP bacteria. Since it is impossible to reproduce drought and sufficient moisture in the field at the same time, we focused on drought and its combination with the herbicide Chistalan and the bacterium *P. protegens* DA1.2. Comparison of similar treatment options in laboratory and field experiments showed similar trends in the content of phytohormones. The amount of ABA decreases in the leaves, and the amount of IAA decreases in the roots after spraying with the herbicide Chistalan. In the same variant of the experiment, the lowest RWC level was indicated. The addition of a culture of *P. protegens* DA1.2 to the herbicide causes RWC reduction to be less significant. However, the use of the bacterium *P. protegens* DA1.2 without herbicide also contributed to a slight decrease in the amount of ABA in the leaves, which was not accompanied by a decrease in RWC. This can be explained by the stimulation of root growth. Thus, the results of the field and laboratory experiments do not contradict each other.

It matters for agricultural practice whether the effects observed at the beginning of vegetation lead to improvement of the harvest. Apparently, there is no definite answer to this question. In our study, the indicators of RWC and hormones worsened in plants after spraying with the herbicide Chistalan. This did not lead to a decrease in the yield or the amount of raw gluten in the grain, but slightly reduced the protein content in the grain. The data obtained can be explained by the suppression of weeds by the herbicide Chistalan, which created favorable conditions for wheat and compensated for the toxic effect of the herbicide. On the contrary, the positive effect of treatment with bacterium *P. protegens* DA1.2 at the tillering stage was combined with both an increase in yield and an increase in the amount of gluten in the grain. The best wheat yield resulting from spraying with a mixture of herbicide and bacterial culture can be associated with a combination of weed control, mitigation of drought, and herbicide stress. There is a relationship between the increase in the yield of the wheat var. Ekada 113 in plots where the bacterium *P. protegens* DA1.2 was used and the activation of tillering, and this relationship led to an increase in the number of productive stems. The data obtained by He et al. [39] indicated the great importance of the amount of auxins and cytokinins in wheat tissues for the initiation of its tillering. However, the effect of bacteria-synthesizing phytohormones on this process still needs to be studied. The results of various studies indicate both an increase in the amount of grain protein, including gluten, under the influence of bacteria [40,41], and the absence of this effect [42], depending on the wheat variety and bacterial strain. Since the amount of grain gluten is important in baking bread, stimulating the accumulation of gluten can be considered a positive property of bacteria.

## 4. Materials and Methods

### 4.1. Materials

The authors of this article isolated bacterium *Pseudomonas protegens* DA1.2 from anthropogenic soil and deposited in the All-Russian Collection of Microorganisms (VCM B-3542D). It is resistant to herbicides and antagonistic to fungi, synthesizes IAA, mobilizes phosphorus, and has nitrogenase activity [18]. The agents for plant treatment were obtained by cultivating bacteria in King B medium [43] in an orbital shaker (Biosan, Riga, Latvia) at a temperature of 28 °C and 160 rpm for 72 h. In order to estimate the counts of *P. protegens* DA1.2 in soil, a serial dilution of soil homogenate in water was used. The number of colony-forming units (CFU) was measured by application to the selective culture medium (g∙L^−1^)—agar-agar, 20.0; Chistalan, 5.0; NH_4_NO_3_, 2.0; MgSO_4_∙7H_2_O, 0.2; KH_2_PO_4_, 2.0; Na_2_HPO_4_, 3; CaCl_2_∙6H_2_O, 0.01; Na_2_CO_3_, 0.1; pH, 7.0. The incubation period at 28 °C was 3 days. The grown colonies and cells of bacteria were compared with colonies and cells of bacterial strain *P. protegens* DA1.2, using a microscope “Leica” DM 1000.

The herbicide Chistalan is selective against dicotyledons (produced by AHK-AGRO LLC, Russia), and contains auxin-like active substances 376 g/L of 2,4-D (2-ethylhexyl ether) and 54 g/L of dicamba (sodium salt).

### 4.2. Laboratory Experiment

When developing the experimental design, three variable parameters were used: soil moisture, herbicide, and inoculation by bacteria. Using these parameters alone or in combination, seven trials were conducted: (1) normal hydration and absence of herbicide and bacteria; (2) normal hydration and spraying with herbicide; (3) normal hydration and inoculation with bacteria; (4) normal hydration and spraying with herbicide and bacteria; (5) moisture deficit and absence of herbicide and bacteria; (6) moisture deficit and spraying with bacteria; (7) moisture deficit and spraying with herbicide; (8) moisture deficit and spraying with herbicide and bacteria. Plants were gown in pots in a light box. The pots were placed on plastic trays according to a completely randomized design, with ten replications.

Wheat (*Triticum aestivum* L.) variety Ekada 113 was planted in 1 L pots with soil mixture and grown under controlled lighting (photon flux density 190 µmol/m^2^/s, a 14 h photoperiod) at a temperature of 22–26 °C. The substrate mixture consisted of 10% washed and screened sand and 90% soil from the arable layer (0–40 cm) of chernozem. Fertilizers were not added to the soil mixture. The seeds were laid out on moistened filter paper. Then ten germinated seeds were planted in each pot. The water content was continuously maintained at 60–80% of soil moisture capacity in the variants with no moisture deficiency. The water deficit corresponded to 30–50% soil moisture throughout the experiment. To determine water losses (evaporation), pots with plants were weighed before watering. The plants were monitored until the end of the tillering phase. Treatment with the herbicide Chistalan and the bacterium *P. protegens* DA1.2 was performed once after the appearance of the third leaf, which is a common practice in the cultivation of spring wheat. A part of each plant was sprayed with an aqueous emulsion (0.2 mL/plant) containing 0.5 mL/L of the herbicide Chistalan, or with a bacterial culture (0.2 mL/plant) diluted with water to a titer of 10^8^ CFU/mL. Some pots were sprayed with herbicide and bacterial culture, mixed in the same amounts. At least 100 plants were used for each type of exposure. The weight of the plants was determined 14 days after treatment. Leaves and roots were weighed on analytical scales HR-250AZG (A&D, Tokyo, Japan) immediately after their separation from the plant.

### 4.3. Soil-Climate Conditions and Experiment Design in the Field

During the two years of the field experiment, drought was a background factor, as there was too little rainfall during the wheat growth period. Therefore, there were no variants with sufficient hydration in the experimental scheme. The study was conducted by the method of randomized subblocks (split-plot), in three repetitions. The herbicide Chistalan was used in the first block, while the second block was left untreated. In subblocks, bacterium *P. protegens* DA1.2 were either used or not.

The field experiment was carried out in 2020 and 2021 at the experimental field of the Ufa Federal Research Centre of the Russian Academy of Sciences (location: Russian Federation, the Urals, latitude 52.60, longitude 58.33, altitude 488 m). The area of each plot was 1000 m^2^, and consisted of Soil-Chernozem Haplic (C_org_ 3.9%, N_tot_ 0.32%, P_Egner_ 143 mg/kg, K_Egner_ 135 mg/kg, pH_KCl_ 6.1). The amount of rainwater and average temperatures during the wheat growth period in 2020–2021 are shown in Table 2.

Bread spring wheat (*Triticum aestivum* L.) of the variety Ekada 113 was grown in the field using agricultural technology recommended for the geographical zone. The predecessor was bread wheat, and 500 seeds per square meter were sown in early May. Mineral and organic fertilizers were not used during the experimental period. Of the pesticides, only the herbicide Chistalan were used, and was sprayed with a manual knapsack sprayer on wheat crops once during the tillering phase. Grain was harvested in August using a combine harvester. The harvest from each plot was stored separately, dried, and weighed. After tillering was over, weeds were counted and identified on 1 m^2^ plots in ten repetitions. Simultaneously, samples were taken for microbiological analysis.

The treatment of the wheat was carried out with the herbicide Chistalan (0.7 L/ha), a suspension of the bacterial strain DA1.2 (2 L/ha with a titer of 10^9^ CFU/mL), or a tank mixture of bacteria and herbicide. Spraying solutions were prepared by mixing herbicide with water in accordance with the recommendations of the producers.

### 4.4. Characteristics of Water Relations

RWC was calculated using the formula of Barrs and Weatherley [44]. In order to determine RWC, the mature first leaves of 10 plants were weighed, their bases were placed in distilled water in a tightly closed vessel to saturate the air with moisture, and placed in darkness at room temperature. After 24 h, the turgid weight (TW) was determined after blotting, and the dry mass was determined after drying for 24 h at 80 °C. Fresh weight (FW), dry weight (DW), and TW were used to determine relative water content: RWC = (FW − DW)/(TW − DW). Evapotranspiration was measured by the weight loss of pots with plants.

### 4.5. Chemical Analyses

The amount of MDA was measured on the seventh day. IAA, zeatin-type cytokinins, and ABA in plant tissues were determined on the second day after spraying the plants. The youngest leaves were used for chemical tests. To isolate phytohormones, shoots and roots were homogenized and extracted with 80% ethanol. Zeatin and its derivatives (in particular, zeatin riboside) in aqueous residue were concentrated on a C-18 cartridge (Waters Corporation, Milford, MA, USA). After solvent evaporation, the dry residue was dissolved in 0.02 mL of 80% ethanol, and zeatin metabolites were separated using thin-layer chromatography [45]. Extraction of ABA and IAA from aliquots of aqueous residues was performed with the diethyl ether, according to the modified method, as described by Kudoyarova et al. [46]. Hormones were immunoassayed using the corresponding specific antibodies.

Membrane lipid peroxidation was assayed as the amount of MDA, a product of lipid peroxidation. Fresh wheat leaves were homogenized in 10% trichloroacetic acid and then centrifuged at 10,000× *g* rpm. The amount of MDA in the extract was determined by the spectrophotometric method, by reaction with thiobarbituric acid [47]. Measurements were carried out in three biological and three analytical repetitions.

### 4.6. Grain Properties

Averaged grain samples were prepared by mixing equal amounts of grain from two harvests. The grain was ground in a laboratory mill with a sieve cell diameter of 0.9 mm. The Kjeldahl method was used to determine the protein content of grain. The protein amount was calculated with a nitrogen conversion factor of 5.7. Gluten was extracted in the following way: dough was prepared using 2% sodium chloride solution at a rate of 60% by weight of flour. The prepared dough was kept in water for 40 min, and was then washed under running water until most of the starch had been washed out and the washing water became clear. The resulting viscoelastic mass was wet gluten.

### 4.7. Statistical Analysis

The data were processed using Statistica (Statsoft) software (version 10). In all figures and tables, data are presented as mean ± standard error. The significance of the differences was assessed by ANOVA, followed by Duncan’s test (*p* ≤ 0.05).

## 5. Conclusions

The results of this study demonstrate that the inclusion of the bacterium *P. protegens* DA1.2 in a Chistalan-containing mixture can improve the growth condition of young wheat plants during dry periods, and can contribute to increasing their yield. At the same time, the auxin-like herbicide used and the auxin-producing bacterium *P. protegens* DA1.2 had different effects on the ratio of phytohormones. As a result, treatment with the strain *P. protegens* DA1.2 could favor the growth of the root system and the provision of wheat plants with water. Due to the use of only one bacterial strain and herbicide, and the limited amount of similar data from the literature on the simultaneous effects of drought, herbicides, and PGP bacteria, the proposed explanation can only be considered to be an assumption. The ability of bacteria to synthesize auxins may be an important feature of PGP to take into account in order to alleviate the negative effects of auxin-based herbicides; however, studies extending to other auxin-producing bacteria should be carried out.

## Figures and Tables

**Table 1:** Relative water content, amount of phytohormones in shoots and roots, and wheat yield parameters in field plots differing in the use of the herbicide Chistalan and the bacterium *Pseudomonas protegens* DA1.2.

	Without Herbicide		Chistalan	
	No Bacteria	Bacteria	No Bacteria	Bacteria
Leaves RWC, %	87.34 ± 1.36 ^b1^	88.05 ± 1.20 ^b^	78.50 ± 0.78 ^a^	86.89 ± 1.28 ^b^
Leaves ABA, ng/g	32.36 ± 2.04 ^d^	27.60 ± 1.97 ^c^	19.58 ± 2.06 ^b^	26.39 ± 2.15 ^c^
Roots IAA, ng/g	16.41 ± 1.54 ^b^	23.07 ± 2.02 ^b^	10.40 ± 1.76 ^a^	15.98 ± 1.57 ^b^
Productive stems, m^−2^	389.3 ± 19.1 ^b^	513.7 ± 16.3 ^c^	357.6 ± 9.6 ^a^	585.7 ± 24.2 ^d^
Weight of 1000 grains, g	33.4 ± 1.7 ^a^	32.0 ± 1.5 ^a^	30.4 ± 1.8 ^a^	30.5 ± 2.0 ^a^
Grains in the spike	16.9 ± 0.8 ^a^	17.7 ± 0.9 ^ab^	19.3 ± 0.7 ^b^	19.8 ± 0.8 ^b^
Yield, kg/ha	2180 ± 143 ^a^	2930 ± 127 ^b^	2090 ± 124 ^a^	3610 ± 132 ^c^
Straw, kg/ha	2200 ± 38 ^a^	3650 ± 81 ^c^	2420 ± 40 ^b^	4370 ± 33 ^d^

^1^ Different letters in each line indicate significant differences between treatments (*p* < 0.05; Duncan’s test).

**Table 2:** Climatic factors in the experimental field during two cropping seasons (2020–2021).

	May	June	July	August	September
	Rainfall, mm				
2020	24.0	0	32.7	15.3	0
2021	0.4	26.2	0.5	4.4	12.9
Average long-term	29.1	43.9	49.4	44.1	28.0
	**Average temperature, °C**				
2020	+16.0	+18.0	+23.1	+20.1	+12.3
2021	+17.2	+19.6	+19.5	+22.2	+8.1
Average long-term	+12.5	+16.8	+18.5	+16.4	+10.2

## Data Availability

Bacteria *Pseudomonas protegens* DA1.2 was deposited in the All-Russian Collection of Microorganisms. Bacteria were assigned a number VCM B-3542D and a description of their properties is available on http://www.vkm.ru ( accessed on 13 April 2021).
